# Down regulated lncRNA MEG3 eliminates mycobacteria in macrophages via autophagy

**DOI:** 10.1038/srep19416

**Published:** 2016-01-13

**Authors:** Kamlesh Pawar, Carlos Hanisch, Sergio Eliseo Palma Vera, Ralf Einspanier, Soroush Sharbati

**Affiliations:** 1 Institute of Veterinary Biochemistry, Department of Veterinary Medicine, Freie Universität Berlin, Oertzenweg 19b, 14163 Berlin, Germany

## Abstract

Small non-coding RNA play a major part in host response to bacterial agents. However, the role of long non-coding RNA (lncRNA) in this context remains unknown. LncRNA regulate gene expression by acting e.g. as transcriptional coactivators, RNA decoys or microRNA sponges. They control development, differentiation and cellular processes such as autophagy in disease conditions. Here, we provide an insight into the role of lncRNA in mycobacterial infections. Human macrophages were infected with *Mycobacterium bovis* BCG and lncRNA expression was studied early post infection. For this purpose, lncRNA with known immune related functions were preselected and a lncRNA specific RT-qPCR protocol was established. In addition to expression-based prediction of lncRNA function, we assessed strategies for thorough normalisation of lncRNA. Arrayed quantification showed infection-dependent repression of several lncRNA including MEG3. Pathway analysis linked MEG3 to mTOR and PI3K-AKT signalling pointing to regulation of autophagy. Accordingly, IFN-γ induced autophagy in infected macrophages resulted in sustained MEG3 down regulation and lack of IFN-γ allowed for counter regulation of MEG3 by viable *M. bovis* BCG. Knockdown of MEG3 in macrophages resulted in induction of autophagy and enhanced eradication of intracellular *M. bovis* BCG.

Mycobacterial species rank among the most potent bacterial pathogens threatening the health of both humans and animals. *M. tuberculosis* is the etiological agent of human tuberculosis and is the leading cause of death from a single infectious bacterial pathogen in humans. One-third of the world’s population is estimated to have latent tuberculosis while multidrug resistance is occurring worldwide (WHO Fact sheet N°104). The vaccination based on using the attenuated strain *M. bovis* BCG (BCG) does not provide enough immunity^1^ and treatment of latent as well as active tuberculosis implies prolonged medication using a combination of therapeutics. Tremendous efforts have been made to investigate the molecular details of host cell response to mycobacterial infections. However, there is still a need for understanding regulatory networks in infection that may lead to new approaches in translational medicine.

Cellular response to bacterial infections relies on well-defined networks of molecular interactions based on regulation of gene expression and protein function. Interestingly, recent RNAseq studies have shown that most of human genome is transcribed but very little of it has the ability to encode proteins^2^. Members of the non-coding genome include microRNA (miRNA) that are a major class of well characterised, conserved and endogenous small interfering RNA that regulate gene expression^3^. Several recent studies pointed out their involvement in mycobacterial infections^4^,^5^.

Another class of regulating non-coding RNA is distinguished from the others primarily based on their size of larger than 200 nt. This class is referred to as lncRNA. Their numbers are estimated to match those of known protein coding genes but they are generally shorter, have fewer exons and possess low evolutionary conservation^6^,^7^. Furthermore, they have lower level of cellular concentration than protein coding transcripts but have higher degree of tissue specificity^8^,^9^. Global expression of lncRNA can be studied e.g. by whole-transcriptome sequencing. Different studies have used RNAseq to study lncRNA expression in different contexts^10^,^11^,^12^. Mentioned tissue specificity, low level of cellular concentrations and lack of understanding on mode of action in most cases raise the question whether a targeted approach based on e.g. RT-qPCR arrays shall be an alternate strategy to study lncRNA in a defined biological context.

LncRNA frequently localise in the nucleus functioning both in *cis* (at the site of their transcription) as well as in *trans* (at the sites on other chromosomes), which points to potential functions as interfaces with the epigenetic machinery, chromatin organisation and regulation of gene expression. They function e.g. as protein scaffolds, activators or inhibitors of transcription, antisense RNA or miRNA sponges, respectively^13^,^14^. The latter has been reported recently as a novel mode of action of lncRNA, where they act as a competing endogenous RNA (ceRNA). This suggests the existence of a network of lncRNA and mRNA, which crosstalk based on mutual miRNA response elements (MRE)^15^. In this context, susceptibility to ceRNA competition has been shown to differ depending on endogenous miRNA:target pool ratios^16^.

Recent studies have demonstrated innate immune related expression of lncRNA following activation of monocytes, dendritic cells or macrophages^7^. Interestingly, a few studies have pointed out the role of lncRNA in regulating autophagy in cancer. For example, it has been shown that decreased expression of the lncRNA MEG3 results in activation of autophagy in bladder cancer cells^17^. However, the molecular details of mode of action are not understood. Rapamycin and other inhibitors of the Ser/Thr protein kinase ‘mammalian target of rapamycin’ (mTOR) are potent inducers of autophagy and inhibitors of phosphatidylinositol 3-kinases (PI3K) such as 3-methyladenine (3-MA) are well-known inhibitors of autophagy^18^. Autophagy is known to be an important antimicrobial process for eradication of *M. tuberculosis* in infected macrophages^19^. However, induction of autophagy during macrophage infection with *M. tuberculosis* or BCG depends on IFN-γ and is enhanced by the presence of 1,25-dihydroxyvitamin D3. This process depends on Beclin1 and Atg5 and therefore constitutes the canonical form^20^. Beclin1 functions as a scaffold for assembly of the PI3KC3 complex to initiate autophagosome formation^21^. Beclin1 was shown to have a role in the convergence between autophagy and apoptosis and to be a substrate of CASP3^21^,^22^. Autophagy and apoptosis are two interconnected cellular processes that are both modulated by pathogenic mycobacteria. In this context, we have reported earlier that in *M. avium* infection of human macrophages inhibition of apoptosis is grounded in let-7e and miR-29a mediated regulation of CASP3 and 7^5^.

This study focuses mainly on two aspects, first the establishment of a RT-qPCR based approach to predict lncRNA function. Secondly, proof of principle by mechanistic studies. We hypothesise that the innate immune response of human macrophages to mycobacterial infections is controlled by lncRNA. Furthermore, we speculate that mycobacterial infection and IFN-γ stimulation of human macrophages leads to lncRNA-mediated induction of autophagy to combat intracellular bacteria. To address this, we preselected a list of lncRNA with known immune related functions and established a customised protocol for arrayed quantification of lncRNA expression based on RT-qPCR.

## Results

### A targeted approach for expression based prediction of lncRNA function

In this study, we aimed at designing a targeted approach based on selecting lncRNA, which are known to have immune related functions and have not been reported in context of mycobacterial infections. Therefore, an RT-qPCR based protocol for individual as well as arrayed quantification of lncRNA was established considering novel aspects for accurate normalisation of lncRNA expression. A list of 21 human lncRNA with immune related functions was compiled using the database lncRNAdb^23^. Initial testing resulted in selection of 17 lncRNA exhibiting harmonised annealing temperatures at 60 °C, dynamic ranges of higher than 8 logs of magnitude and mean efficiency of 96.62% ± 4.42% (Table 1).

**Figure 1:**
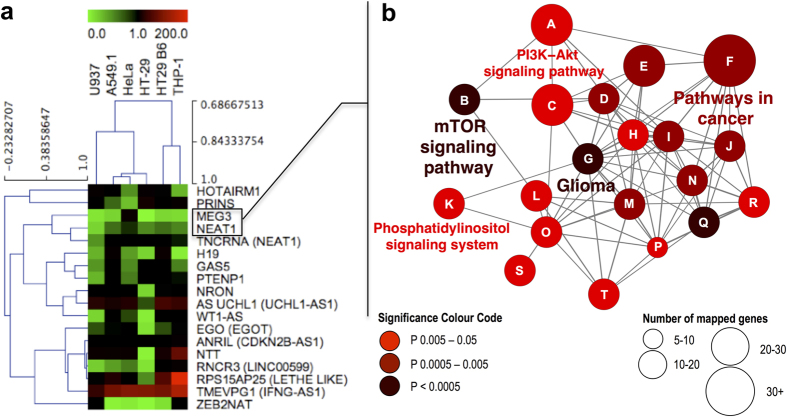
Hierarchical clustering of lncRNA expression and prediction of pathways. (**a**) Expression of selected lncRNA was studied in six cell lines and mean fold changes of expression vs. a common reference were hierarchically clustered in a heat map (decreased expression is shown in green and increased in red according to the colour bar shown above). All experiments were performed at least in triplicates. B2M and ACTB were used as reference genes. (**b**) *In silico* analysis assigned the cluster consisting of MEG3 and NEAT1 to mTOR and PI3K-AKT signalling pathways as indicated by the ClueGO network. Colours represent the P value of significant enrichment and size of circles describes number of mapped genes as indicated in the legend. Legend: A, PI3K-AKT signalling pathway; B, mTOR signalling pathway; C, Rap1 signalling pathway; D, HIF-1 signalling pathway; E, protoglycans in cancer; F, pathway in cancer; G, glioma; H, melanoma; I, prostate cancer; J, FoxO signalling pathway; K, Phosphatidylinositol signalling system; L, insulin signalling pathway; M, ErbB signalling pathway; N, pancreatic cancer; O, estrogen signalling pathway; P, endometrial cancer; Q, chronic myeloid leukaemia; R, colorectal cancer; S, adregenic signalling pathway; T, neutrophin signalling pathway.

Epithelial as well as immune cell lines provide widely recognised *in vitro* models for addressing different aspects of interactions between bacterial agents and host cells and help to reduce animal experiments. We applied a set of human monocytic (THP-1 and U937) as well as epithelial cell lines (A549.1, HeLa, HT-29 and HT-29/B6), which serve as common *in vitro* infection models and evaluated the performance of the presented approach. Furthermore, we aimed at developing a strategy for functional prediction and pathway annotation of selected lncRNA. Cell line specific expression was examined and lncRNA-clusters were deduced from determined expression patterns that were subjected to functional predictions. For this purpose, lncRNA expression in investigated cell lines (fold changes of a given sample vs. a common reference) was hierarchically clustered. Distinct groups of lncRNA such as H19, PTENP1 and GAS5 or MEG3 and NEAT1 were defined, which showed matched expression among cell lines and exhibited small distances in hierarchy (Fig. 1a). Identified clusters were chosen for prediction and annotation of molecular pathways. We took advantage of the evolving concept that lncRNA, mRNA and pseudogenes talk to each other based on mutual MRE^15^. Consequently, CLIP-Seq based interactions of lncRNA and miRNA were obtained from starBase^24^ to determine miRNA that target lncRNA of an identified cluster (Fig. 1a and supplementary information). The resulting list of miRNA was subjected to target analysis and a network of mRNAs was generated that were inferred to cross talk to the lncRNA via mutual MRE. For this purpose, we employed Cytoscape^25^ together with its app CyTargetLinker^26^ and predicted miRNA-mRNA networks by considering regulatory interaction networks of target scan^27^. The resulting target networks (mRNA sharing MREs with lncRNA) of an identified lncRNA cluster (Fig. 1a and supplementary information) were intersected and subjected to ClueGo analysis^28^ for determining significant KEGG^29^ pathway enrichment (detailed information on the approach is provided in the supplementary information). For example, the cluster consisting of H19, PTENP1 and GAS5 was mainly assigned to gene ontologies ‘specific DNA binding’ and ‘protein kinase activity’ and genes were enriched in signalling pathways such as FoxO, HIF-1 or Estrogen (supplementary information). Our analysis pointed out a particular cluster with small distance among the members NEAT1 and MEG3. The introduced prediction strategy assigned this cluster inter alia to mTOR (13 genes; 22% genes/term; P < 0.01) and PI3K-AKT (30 genes; 9% gene/term; P < 0.05) signalling pathways, which are involved in regulation of autophagy (Fig. 1b and supplementary information).

### lncRNA are distinctly down regulated in early BCG infection of macrophages

In an earlier study, we have shown that macrophage apoptosis in *M. avium* infection of human macrophages is regulated by miRNA^5^. Based on pathway predictions presented above (mTOR and PI3K-AKT) and considering the idea of a miRNA based common language^15^, we hypothesised that lncRNA play a substantial role in mycobacterial infections. For approaching this issue, we adjusted the established lncRNA RT-qPCR to a protocol that allowed for arrayed and customised quantification^30^. This layout enabled the detection of 17 individual lncRNA (preselected from initial individual infection experiments) within a single RT-qPCR. 5 additional references (SNORD47, B2M, ACTB, GAPDH and 18S rRNA) were considered for normalisation of lncRNA expression. For this purpose, THP-1 derived macrophages were infected with viable BCG as well as BCG heat killed (hk) and non infected cells served as negative controls.

TLR4 activation of macrophages has been shown to cause early, intermediate and late waves of transcription and the early response has been proposed to be important for the regulation of later events^31^. Moreover, modulation of host macrophage defence mechanisms takes place as early as 10 min after infection. For example, Rab5 is recruited to the BCG containing phagosome at 10 min post infection (p.i.)^32^ and secreted mycobacterial proteins accumulate as early as 30 min after infection in cellular compartments of host macrophages^33^. Several mycobacterial components such as lipoarabinomannan (LAM) are known to interfere with and manipulate the regulation of early host cell response. Therefore, we selected the time points 30 min and 4 h for monitoring lncRNA regulation of early events.

**Figure 2:**
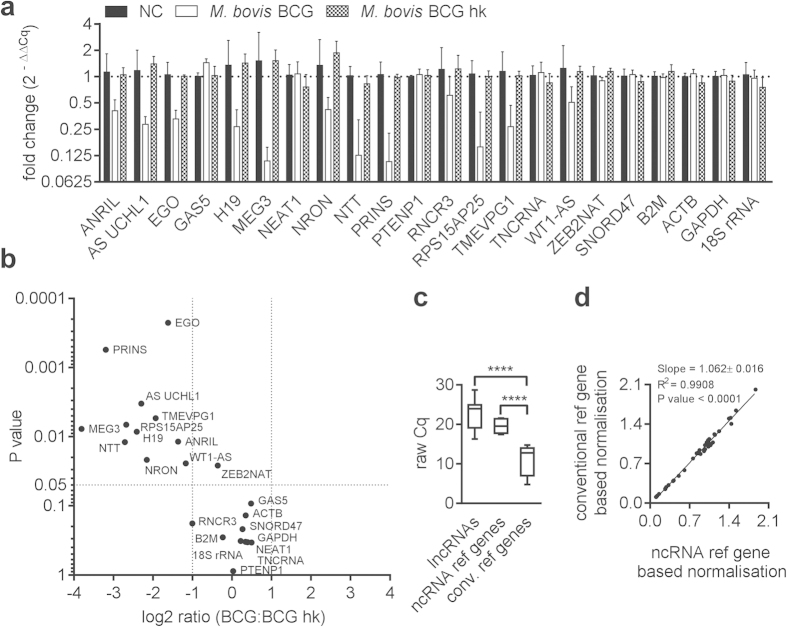
lncRNA expression and normalisation at 30 min p.i. of macrophages infected with viable and heat killed *M. bovis* BCG. (**a**) In total, 11 lncRNA showed clearly decreased expression after infection with viable BCG when compared with BCG hk. Columns show mean fold change between samples and non infected controls (NC) of three biological replicates, each measured in triplicates, while bars indicate SD. ZEB2NAT, SNORD47 and PTENP1 were used as reference genes. (**b**) The plot shows significantly (unpaired t test) dysregulated lncRNA in BCG infected macrophages compared with BCG heat killed (hk) treated cells. Dotted lines indicate statistically significant dysregulation (P < 0.05, log2 ratio <−1 or >1). (**c**) After identification of most stable genes by means of geNorm analysis two groups of reference genes and a remaining group were defined: ncRNA references (ZEB2NAT, SNORD47 and PTENP1), conventional references (B2M, GAPDH and ACTB) and lncRNAs (remaining 15 lncRNA). Box plots show raw Cq values of groups (****P < 0.0001, unpaired t test). (**d**) lncRNA expression was normalised with the geometric mean of either conventional references or ncRNA references and data were plotted to determine the coefficient of determination (R^2^), slope and Y-intercept.

Several lncRNA turned out to be significantly down regulated in early stages of infection (30 min) compared with BCG hk (Fig. 2a,b). This observation was made only in infections with viable BCG in contrast to BCG hk treated cells as well as non infected controls (Fig. 2a). As shown in Fig. 2b, a total of 11 lncRNA showed significant and higher than twofold decreased expression after infection with viable BCG compared with BCG hk (log2 ratio _BCG:BCG hk_ <−1). To name a few, NTT exhibited 7-fold, PRINS 9-fold and TMEVPG1 4-fold significantly decreased expression (Fig. 2b). Among all down regulated lncRNA at 30 min, MEG3 showed most pronounced and significantly decreased expression having more than 10-fold lower values compared with BCG hk (Fig. 2b, unpaired t test, P < 0.01).

**Figure 3:**
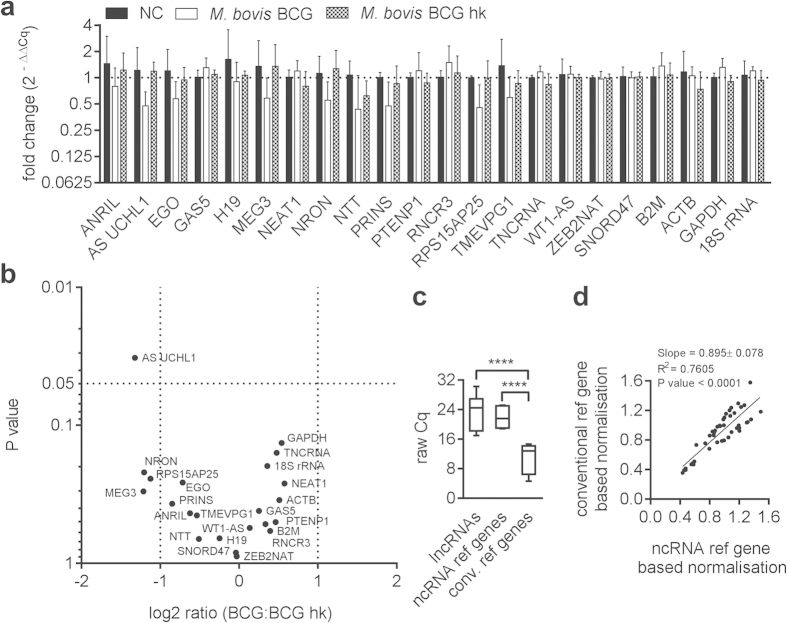
lncRNA expression and normalisation at 4 h p.i. of macrophages infected with viable and heat killed *M. bovis* BCG. (**a**) A trend of decreased expression of several lncRNA was determined after infection with viable BCG at 4 h p.i. compared with BCG hk. Columns show mean fold change between samples and non infected controls (NC) of three biological replicates, each measured in triplicate, while bars indicate SD. ZEB2NAT, SNORD47 and WT1-AS were used as reference genes. (**b**) The plot shows only one significantly (unpaired t test) dysregulated lncRNA in BCG infected macrophages compared with BCG heat killed (hk) treated cells. Dotted lines indicate statistically significant dysregulation (P < 0.05, log2 ratio <−1 or >1). (**c**) After identification of most stable genes by means of geNorm analysis two groups of reference genes and a remaining group were defined: ncRNA references (ZEB2NAT, SNORD47 and WT1-AS), conventional references (B2M, GAPDH and 18 S rRNA) and lncRNAs (remaining 15 lncRNA). Box plots show raw Cq values of groups (****P < 0.0001, unpaired t test). d) lncRNA expression was normalised with the geometric mean of either conventional references or ncRNA references and data were plotted to determine the coefficient of determination (R^2^), slope and Y-intercept.

At 4 h p.i., a similar trend of lncRNA dysregulation was observed in infection experiments comparing viable BCG with BCG hk. However, only AS UCHL1 showed significant and more than twofold down regulation (Fig. 3a,b).

### Assessment of strategies for normalisation of lncRNA expression

lncRNA are known to exhibit lower cellular expression levels compared with protein coding genes. So we raised the question whether normalisation of lncRNA expression by means of conventional reference genes (mRNA) is advisable. To approach this, we tested the application of two sets of references: One set consisted of 3 stably expressed conventional reference genes and another set included stably expressed ncRNA. Except for the studied 17 lncRNA, we considered one small nuclear RNA (SNORD47) as well as 3 conventional reference mRNA (B2M, ACTB and GAPDH) together with 18 S rRNA for evaluating normalisation strategies of lncRNA expression. Two successive rounds of geNorm analysis^34^ were performed (considering the total number of 22 targets) to identify the most stable genes among both the conventional references and the entire list of ncRNA. At 30 min p.i., B2M, GAPDH and ACTB turned out to be stably expressed as represented by low geNorm M values (M_B2M_ = 0.503, M_ACTB_ = 0.557, M_GAPDH_ = 0.521). Furthermore, geNorm analysis confirmed a group of three ncRNA including ZEB2NAT, SNORD47 and PTENP1 to be stably expressed (M_ZEB2NAT_ = 0.487, M_SNORD47_ = 0.565, M_PTENP1_ = 0.581), which were chosen as ncRNA references. To evaluate differences in cellular abundance of studied RNA classes, raw Cq values of lncRNA were compared with identified ncRNA references as well as conventional references. As shown in Fig. 2c, the mean raw Cq value of conventional reference genes was significantly lower compared with the selected ncRNA reference genes and remaining lncRNA (Mean Cq _conv. ref genes_ = 11.2, Mean Cq _ncRNA ref genes_ = 19.5, Mean Cq _lncRNAs_ = 22.9, unpaired t test P < 0.0001). Consequently, the geometric mean of either reference ncRNA or conventional reference genes was used to normalise lncRNA expression. Obtained sets of data were plotted to determine the correlation. Strong linearity was observed between both sets of data as shown by the calculated values: slope = 1.062 ± 0.016, R^2^ = 0.9908 and Y-intercept = −0.02921 ± 0.01515 (Fig. 2d). Although both sets of references were examined to be stable and suitable for normalisation of lncRNA expression, the distinct differences in cellular abundance (Fig. 2c) call the use of conventional reference genes into question.

At 4 h p.i., geNorm analysis resulted in lower M values for the reference ncRNA (M_ZEB2NAT_ = 0.536, M_WT1-AS_ = 0.541, M_SNORD47_ = 0.579) compared with the conventional references (M_18_ _S rRNA_ = 0.561, M_GAPDH_ = 0.617, M_B2M_ = 1.0). The raw Cq values were again significantly lower in the conventional reference genes compared with the other two groups (reference ncRNA and lncRNA) supporting the observations that were made above (Fig. 3c: Mean Cq _conv. ref genes_ = 11.1, Mean Cq _ncRNA ref genes_ = 21.9, Mean Cq _lncRNAs_ = 23.4, unpaired t test P < 0.0001). As shown in Fig. 3d, the linear relationship between two data sets was not as pronounced as calculated above (slope = 0.895 ± 0.078, R^2^ = 0.7605 and Y-intercept = 0.05127 ± 0.0769).

### Induction of autophagy by IFN-γ in BCG infected macrophages accounts for sustained MEG3 down regulation

**Figure 4:**
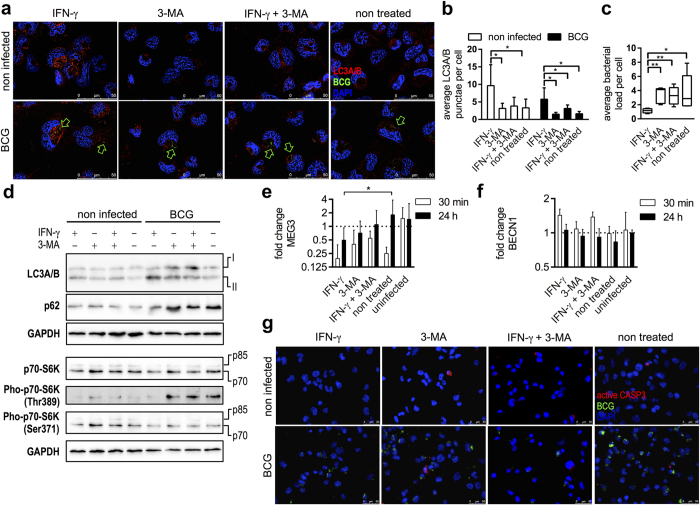
Induction of autophagy is associated with MEG3 down regulation without activation of Caspase 3. (**a**) Autophagy in BCG infected cells and non infected controls (NC) was induced or inhibited with IFN-γ or 3-MA, respectively. Autophagy was tracked by detecting the marker LC3A/B (red) in macrophages. Green arrows indicate fluorescently labelled BCG (green, indicated by arrows). Nuclei were stained with DAPI (blue). Scale bars indicate 50 μm. (**b**) LC3A/B punctae were quantified using ImageJ. IFN-γ treatment increased the average number of LC3A/B punctae in both infected cells and non infected controls (unpaired t test, P < 0.05). (**c**) Number of intracellular BCG was significantly diminished in IFN-γ treated macrophages (unpaired t test, P < 0.05). (**d**) Autophagy and mTOR activation was studied in infected and control cells by means of western blots. BCG infection along with IFN-γ induced autophagy was examined by LC3A/B conversion and p62 degradation. mTOR activation was reduced in same samples as shown by clearly decreased p70-S6K (Thr389) phosphorylation. GAPDH is shown last as respective loading reference. (**e,f**) MEG3 and Beclin1 expressions were followed at 30 min and 24 h p.i. along with induction and inhibition of autophagy. IFN-γ treatment caused consistent and significant down regulation of MEG3 in infected cells compared with non treated controls (unpaired t test, P < 0.05). There was no significant change of Beclin1 expression. Columns show mean fold changes over non infected controls of at least three biological replicates normalised with SNORD47, each measured at least in triplicate. Bars indicate SD. (**g**) Induction of apoptosis was tracked by detecting active CASP3 (red). BCG was fluorescently labelled (green) and nuclei counterstained with DAPI (blue). Scale bars indicate 50 μm.

Autophagy is a cellular process for degradation and recycling of considerable cytoplasmic particles. Along these lines, recent studies have elucidated autophagy as a cell-autonomous defence mechanism for eradication of intracellular bacterial pathogens such as *Salmonella*, *Listeria*, *Shigella* and pathogenic *Mycobacterium* species^19^. It has been shown recently that MEG3 knock down in bladder cancer cells resulted in induction of autophagy, increased cell proliferation and inhibition of apoptosis^17^. For studying the effects of autophagy on MEG3 levels in the context of BCG infection, we inhibited or activated autophagy in macrophages by addition of 3-MA or IFN-γ, respectively. IFN-γ has been shown to be a potent inducer of autophagy in BCG infected macrophages^35^. Autophagy was examined by detecting the autophagosomal marker LC3A/B. As shown in Fig. 4a,b, IFN-γ treatment along with BCG infection resulted in an increase of LC3A/B punctae and 3-MA compensated for it. The quantification of LC3A/B punctae proved IFN-γ as potent inducer of autophagy in macrophages both in infected and non infected samples (Fig. 4b, unpaired t test P < 0.05). However, BCG without IFN-γ treatment was not sufficient to increase the number of punctae to the same extent. Interestingly, IFN-γ treatment resulted in efficient eradication of BCG as proven by significant decrease (unpaired t test, P < 0.05) of green fluorescence signals, while in other treatments sustained green fluorescence was detected over the course of experiment (Fig. 4c). Induction of autophagy was proved by LC3A/B conversion and p62 degradation in infected and IFN-γ treated macrophages compared with the controls (Fig. 4d). To determine effects of treatments on mTOR activation, p70-S6K phosphorylation was studied. Infection alone or along with 3-MA treatment caused mTOR activation shown by phosphorylation of p70-S6K (Thr389). However, treatment with IFN-γ along with infection distinctly decreased phosphorylation of Thr389 proving mTOR inactivation. Also phosphorylation of the residue Ser371 declined after treatment (Fig. 4d). In the same set of experiments RNA was isolated to determine MEG3 and Beclin1 expression. Beclin1 is a key regulator of autophagy and has been described recently as an interface between apoptosis and autophagy^21^. Induction of autophagy by IFN-γ in infected macrophages was accompanied by sustained and significant (unpaired t test P < 0.05) MEG3 down regulation (over 24 h), while 3-MA administration partly restored MEG3 expression (Fig. 4e). Combinations of both resulted also in partially restored MEG3 expression accompanied by inhibition of autophagy (Fig. 4d). BCG infected and non treated macrophages exhibited clearly decreased MEG3 levels at 30 min p.i., however, MEG3 expression converged normal levels at 24 h. The treatments had no pronounced effects on Beclin1 expression (Fig. 4f, unpaired t test P > 0.05). To rule out apoptotic effects on our observations, active CASP3 was examined by immunofluorescence. There was no obvious and general activation of CASP3 in any case of treatment (Fig. 4g).

### MEG3 knockdown induces LC3A/B conversion in macrophages

**Figure 5:**
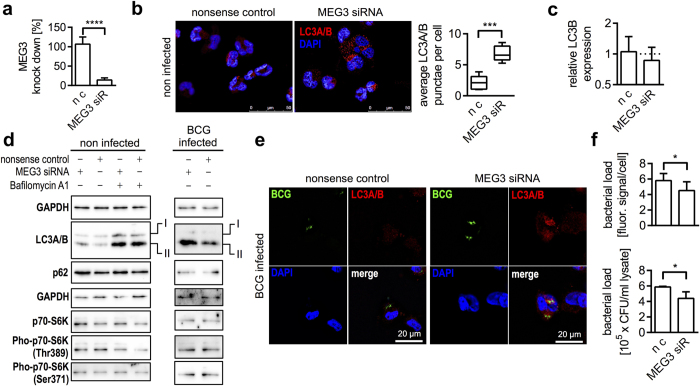
MEG3 knockdown induces autophagy. (**a**) Knockdown in THP-1 using a specific siRNA resulted in significantly (unpaired t test, P < 0.0001) decreased cellular MEG3 levels compared with nonsense-transfected control(n c). Experiments were repeated at least 3 times. (**b**) MEG3 knockdown resulted in significantly (unpaired t test, P < 0.001) increased LC3A/B punctae compared with nonsense transfected controls (n c). Box plots show at least three biological replicates. (**c**) MEG3 knockdown had no effect on LC3B transcriptional levels. (**d**) MEG3 knockdown triggered autophagy as demonstrated by increased LC3A/B conversion in both infected and non infected macrophages. mTOR activation was determined by evaluating p70-S6K phosphorylation. GAPDH is shown first as respective loading reference. (**e**) After knockdown LC3A/B punctae were co-localised with intracellular BCG d) MEG3 knockdown significantly decreased intracellular BCG numbers as determined by quantification of fluorescence signals as well as CFU (unpaired t test, P < 0.05).

To determine whether MEG3 is a regulator of autophagy in macrophages, we efficiently knocked down MEG3 (Fig. 5a) and tested effects on autophagy by quantification of LC3A/B punctae per cell as well as by means of western blots examining its conversion. The application of siRNA potently decreased (unpaired t test, P < 0.0001) cellular levels of MEG3 in THP-1 derived macrophages and clearly increased LC3A/B punctae as represented by immunofluorescence detection (Fig. 5b). Quantification resulted in significant increase of LC3A/B punctae after MEG3 knockdown compared with nonsense transfected controls (Fig. 5b, unpaired t test P < 0.001). MEG3 knockdown had no obvious effects on LC3B mRNA expression (Fig. 5c) and LC3A levels were under the detection limit.

In a guideline for autophagy detection, investigators were made aware of efficiency of transfections, because a western blot will detect LC3A/B in the entire cell population, including those that are not transfected^36^. The transfection resulted in more than 80% knockdown of MEG3 in THP-1 compared with nonsense transfected controls (Fig. 5a). As shown in Fig. 5d, MEG3 knockdown increased LC3A/B conversion compared with nonsense transfected controls as shown by enhanced intensity of LC3A/B-II in western blots. Addition of Bafilomycin A1 accumulated LC3A/B levels without clear differences between samples and controls. However, there was no obvious degradation of p62 after siRNA treatment in non infected cells. The levels of p62 slightly increased in MEG3 siRNA treated cells (Fig. 5d). To determine whether MEG3 knockdown has effects on mTOR activation, we detected p70-S6K phosphorylation. MEG3 knockdown resulted in increased total cellular levels of p70-S6K in both Bafilomycin A1 treated and untreated cells compared with nonsense controls. P70-S6K (Thr389) phosphorylation was slightly decreased after MEG3 knockdown indicating mTOR inactivation (Fig. 5d). Knockdown had no effect on phosphorylation of the residue Ser371 (Fig. 5d). Moreover, MEG3 knockdown along with BCG infection resulted in clearly increased cellular LC3A/B-II levels compared with controls and p62 levels were slightly increased in MEG3 siRNA and infected controls (Fig. 5d). RNAi resulted in accumulation of LC3A/B signals that were co-localised with BCG (Fig. 5e), which went along with enhanced eradication of intracellular BCG as examined by two independent methods (detection of fluorescently labelled BCG as well as CFU). Both methods revealed significantly decreased bacterial loads after MEG3 knockdown in THP-1 derived macrophages compared with nonsense controls (Fig. 5f, unpaired t test P < 0.05).

## Discussions

Currently, about 9000 annotated human genes are known to produce more than 14000 lncRNA transcripts^37^. Although dysregulation of lncRNA has been reported recently in different aspects of innate immune response^7^, the function of majority of these transcripts is yet to be determined. The mentioned tissue specific expression of lncRNA suggests the application of well-established cell lines as suitable models for studying molecular functions of lncRNA in a particular biological process. For this and other purposes, we developed a targeted approach for RT-qPCR based expression analysis of lncRNA. The employed layout based on 96 well plates is extendable to include a total number of 26 lncRNA together with 5 reference RNAs each measured in triplicates. To our knowledge, this is the first non-commercial approach for arrayed RT-qPCR based quantification of lncRNA, which is fully scalable and automatable. We evaluated the application of both conventional and ncRNA reference genes for normalisation of lncRNA expression. A set of three ncRNA was considered for normalisation of lncRNA expression because they exhibited enhanced stability of expression compared with the conventional reference genes. More to the point, there were distinct and significant differences in cellular abundance of conventional references and ncRNA. Diverging cellular abundance of a reference gene and the gene of interest may bias the analysis based on differences in e.g. cellular turn over, RNA integrity or efficiencies of the enzymatic reaction as it has been discussed in the case of 18 S rRNA^38^. We conclude that the application of a stability-tested set of ncRNA is indicated for thorough normalisation of lncRNA expression.

LncRNA constitute a heterogeneous class with low degree of evolutionary conservation and diverse modes of action^39^. This fact aggravates functional predictions and pathway involvement as it has been developed for miRNA and mRNA, respectively. However, in recent years the concept of a common RNA language has been developed, where shared MRE are suggested to serve as letters between different classes of RNA^15^. It has been shown that miRNA-ceRNA interaction depends on cellular ratios of both RNA classes, which aggravates a generalised model for miRNA sponges^16^. However, lncRNA have been reported to have several other modes of action. Our interpretation of the mentioned model considers lncRNA not just as miRNA decoys. LncRNA are known to function as protein scaffolds and are supposed to bind and control transcriptional coactivator or corepressor complexes^40^. Hence, we speculate that miRNA are able to indirectly influence cellular availability of mRNA by targeting e.g. lncRNA that are capable of stimulating or inhibiting transcription. The rationale of the study was to provide an expression-based approach that functionally assigns lncRNA clusters to molecular pathways and enables prediction of lncRNA function by considering lncRNA-miRNA-mRNA crosstalk. As we were analysing lncRNA clusters that were deduced from cell line specific expressions, we came across a particular cluster with small distance between NEAT1 and MEG3. Interestingly, *in silico* analysis linked them to mTOR and PI3K-AKT signalling pathways that are important for regulation of autophagy. For reducing the complexity of the study, we decided to focus only on mechanistic studies of MEG3. This does not necessarily rule out the fact that other investigated lncRNA may have different or cooperative functions. The data presented here provides a basis for our future research.

Infection of THP-1 derived macrophages with viable BCG caused remarkable dysregulation of several lncRNA. For example, NTT, PRINS, TMEVPG1 and MEG3 were significantly down regulated at 30 min p.i. compared with BCG hk stimulated cells but not at 4 h p.i. We conclude that the pronounced down regulation at 30 min p.i. points to specific functions in responding to physiologically active BCG rather than effects being relevant to mechanisms of uptake. None of the above mentioned lncRNA has been reported in the context of mycobacterial infection. However, recent studies have shown that herein investigated and dysregulated lncRNA are associated with IFN-γ signalling, NF-κB, apoptosis and autophagy^17^,^41^,^42^. NTT was 7-fold down regulated after infection. NTT is a single-copy gene residing in chromosome 6q23-q24, close to the IFN-γ receptor gene and its transcripts were found in activated, but not resting T cells^43^. NTT has been suggested to act as a regulatory RNA by activating PKR, which induces the degradation of I-κBβ with subsequent NF-κB activation^41^. PRINS showed 9-fold decreased expression in BCG infected macrophages. PRINS has been reported to regulate the antiapoptotic effects of the interferon-inducible gene IFI6 in psoriasis^44^. TMEVPG1 was 4-fold down regulated and is located in a cluster of cytokine genes including IFN-γ. TMEVPG1 transcription is dependent upon NF-κB while it contributes to IFN-γ expression as part of the Th1 differentiation program^45^,^46^. AS UCHL1 (antisense UCHL1) showed consistent down regulation at 30 min as well as at 4 h p.i. UCHL1 will be a hot candidate for our future research, since it is the antisense product of the gene UCHL1 and its dysregulation has been related to defects in autophagy in diabetes mellitus^47^.

BCG infection caused effective down regulation of MEG3, which is a tumour suppressor gene located in chromosome 14q32 that has been associated with carcinogenesis^48^,^49^. MEG3 is down regulated in case of human tumours including hepatocellular cancers, meningiomas, bladder cancer and gastric cancer^17^,^50^,^51^,^52^. The reason behind loss of MEG3 expression in tumours has been linked to hyper methylation of the promoter or differentially methylated regions upstream of the MEG3 gene^48^,^50^. There are no reports about involvement of MEG3 in any case of infection. However, decreased expression of MEG3 has been reported to activate autophagy in bladder cancer cells^17^. It is noteworthy to mention that several *M. tuberculosis* products, such as PI3P phosphatase SapM, glycosylated phosphatidylinositol-mimic LAM and phosphatidylinositol mannoside (PIM) have been reported to be able to suppress PI3K signalling and to counteract autophagy^53^. In line with this, BCG has been shown to oppose and inhibit induction of autophagy in macrophages while *M. smegmatis* was devoid of this characteristic^54^. It will be interesting to examine whether mycobacterial products are capable of directly manipulating cellular MEG3 levels. Induction of autophagy by rapamycin mediated inhibition of mTOR signalling has been shown to unblock phagosomal maturation in *M. tuberculosis* and BCG infection^35^. More recent studies have reported IFN-γ and vitamin D to be needed *in vivo* for Beclin 1 dependent autophagy and antimycobacterial activity of macrophages^19^,^20^. Beclin1 is a key regulator of autophagy and an interface between apoptosis and autophagy. CASP3 mediates the cleavage of Beclin1 and produced fragments are devoid of autophagy inducing capacities. However, the C-terminal cleavage product seems to be capable of triggering the release of proapoptotic factors^21^. Consequently, we tested for activation of executioner CASP3 to ensure apoptosis was not triggered. On one hand, IFN-γ induced autophagy in infected macrophages was associated with sustained down regulation of MEG3 over 24 h, while 3-MA partly compensated for the dysregulation. The absence of IFN-γ involved early decreased MEG3 expression, which was restored back to normal over 24 h pointing to a potential counter regulation by BCG. On the other hand, IFN-γ treatment along with BCG infection efficiently compromised p70-S6K (Thr389) phosphorylation indicating mTOR inactivation.

Specific knockdown of MEG3 had no effect on LC3B transcription. Therefore, increasing number of LC3A/B punctae per cell is unlikely to depend on MEG3 mediated expressional changes of LC3A/B. In line with this, increased LC3A/B conversion was observed after MEG3 knockdown in both infected and non infected macrophages. However, RNAi caused no obvious degradation of p62 in macrophages. In MEG3 knockdown cells the ratio of LC3A/B-II to LC3A/B-I increases. This can occur because of increased formation of autophagosomes, or decreased degradation of autophagosomal material in lysosomes. As shown in Fig. 5d, the levels of p62, the autophagy substrate and adaptor for intracellular bacteria, seem to increase in MEG3 siRNA treated cells. This would be consistent with a block in lysosomal degradation and not an increase in autophagosome formation. Phosphorylation of p70-S6K (Thr389) was subtly different in non infected cells. It has been reported that p62 associates with Rags in lysosomes and is required both for the interaction of mTOR with Rag and for translocation of the mTORC1 complex to the lysosome, which is important for mTOR activation^55^. Inhibition of mTOR on the other hand induces autophagy^56^. Taken together, we conclude that MEG3 seems to block lysosomal degradation and has limited effects on mTOR activation. Our results show that IFN-γ mediated induction of autophagy in BCG infection happens in a MEG3 dependent manner. However, other lncRNA may have cooperative effects on autophagy and mTOR activation and will be a focus of our future research. Furthermore, repression of the lncRNA MEG3 results in enhanced eradication of intracellular mycobacteria. We speculate that in absence of IFN-γ, intracellular BCG are able to counter regulate early MEG3 dysregulation resulting in inhibition of autophagy. Presence of IFN-γ, on the other hand, allows for induction of autophagy by sustained MEG3 down regulation reinforcing the eradication of mycobacteria in infected macrophages.

## Methods

### Bacterial cultivation, cell culture and RNA isolation

*M. bovis* BCG (DSMZ No. DSM 43990) was grown as described previously^57^. For microscopy, BCG was fluorescently labelled with 5-(6)-carboxyfluorescein-succinylester (Sigma Aldrich) as described previously^58^.

Human acute monocytic leukaemia cell line THP-1 (DSMZ No. ACC 16), human cervix carcinoma cell line HeLa (ATCC No. CCL-2), human monocytic cell line U937 (DSMZ No. ACC 5), human type II pneumocyte cell line A549.1 (ATCC No. CCL 185), human colorectal adenocarcinoma cells HT-29 (DSMZ No.: ACC 299) and its sub clone HT-29/B6^59^ were cultured as described previously^5^,^57^,^60^,^61^ or following the protocols for nucleofection (Lonza). Total RNA from cell lines was isolated and quality controlled as described earlier^62^.

### Selection of lncRNA, oligonucleotides and RT-qPCR

A total of 17 known human lncRNA molecules were chosen from the lncRNAdb^23^ by using search strings to extract entries with immune related functions. lncRNA sequences were acquired from the same database. We also included a set of 5 non-coding as well as conventional reference mRNA for normalisation (Table 1). All oligonucleotides were synthesised by Sigma Aldrich. A list of lncRNA including primer sequences and amplicon sizes is given in MEP_L_tbl1Table 1.

The cDNA was synthesised from individual samples using the Maxima First Strand cDNA synthesis kit (Thermo Scientific). Pooled cDNA was taken as template for testing the primers listed in Table 1 via gradient RT-qPCR. Amplicons showing the expected size were isolated and sequenced to ensure specificity. Serial dilutions of cDNA pools spiked with specific amplicons (3 μM to 3 fM) were used to determine the dynamic range, efficiency, slope, Y-intersect and R^2^ of lncRNA RT-qPCRs (Table 1). Expression analysis was performed by means of SYBR Green detection chemistry using the SensiMix SYBR Hi-ROX Kit (Bioline GmbH) and the StepOnePlus Cycler (Life Technologies). All reactions were carried out using clear MicroAmp® Fast 96-Well Reaction Plates (Life Technologies) that were sealed with adhesive films. Real-time amplification was carried out via the first step at 95 °C for 10 min, followed by 40 cycles with 15 s at 95 °C, 20 s at 60 °C and 30 s at 72 °C. The fluorescence signal was acquired at 60 °C. Quality control was performed by subsequent melting curve analysis by 95 °C for 15 s, 60 °C for 1 min, ramping from 60 °C to 95 °C at 5 °C/min and acquiring the signal. RT-qPCR experiments and analysis of data were performed according to the MIQE guidelines^63^.

Individual expression analysis of lncRNA was examined in 10 μl final reaction volume. Triplicate measurements were performed using a master mix containing 2× SensiMix SYBR Hi-ROX with 0.1 μM of each forward and reverse primer. After dispensing 9 μl of master mix in respective sample wells in a 96 well plate, 1 μl of a 1:5 dilution of respective RT-reaction was added. The amplification cycles and melting curves were carried out as mentioned above. Samples containing water instead of RT-reaction served as negative controls. Expression of lncRNA in each cell line was compared with a common reference composed of an *ana partes aequales* pool of all investigated RNA samples.

Arrayed RT-qPCR of lncRNA along with reference RNA was performed based on the same mentioned principle. A master mix was prepared in a 675 μl volume consisting of 2× SensiMix SYBR Hi-ROX and 20 μl of non diluted RT-reaction. After dispensing 9 μl of the master mix in wells of a 96 well plate, 1 μl of a 2 μM primer pool consisting of the respective lncRNA specific forward and reverse primer was added to corresponding wells of the array. The amplification cycles and melting curves were carried out as mentioned above. Arrayed quantification was performed in triplicate.

Normalisation of lncRNA expression was performed with a set of reference RNA using the geNorm algorithm^34^. A first round of geNorm analysis resulted in the selection of 10 most stable targets that were subjected to a second round of geNorm analysis to define the most stable 3. Relative quantification (ΔΔCq) was determined after calculation of the difference between ΔCq _sample_ and ΔCq _calibrator_. Fold differences were determined and corrected for efficiency of reactions by calculating 
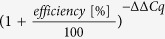
.

### Infection experiments

Monocytic THP-1 cells were seeded without gentamycin in 6 well plates at a density of 1 × 10^6^ cells per well. Cells were differentiated into macrophages by adding 10 nM phorbol-12-myristate-13-acetate (PMA, Sigma Aldich). PMA was removed after 24 h of incubation by washing the cells with warm PBS three times. Cells were incubated for another 24 h in RPMI 1640 supplemented with FBS Superior (Biochrom). Afterwards, macrophages were infected with viable as well as hk BCG at a multiplicity of infection (MOI) of 10. Synchronised infections^64^ were performed by cooling down to 4 °C for 10 min followed by addition of mycobacteria and another incubation at 4 °C for 10 min. Afterwards, cells were immediately transferred to the incubator and warmed up to 37 °C and 5% CO_2_. Non infected cells were treated equally and served as a control. Samples were taken at 30 min and 4 h for RT-qPCR. For immunostaining, cells were infected as described earlier^57^ and samples were taken at 24 h. All experiments were performed at least in triplicate.

### Induction and inhibition of autophagy

3-MA (R & D Systems) is an inhibitor of PI3K. 3-MA inhibits autophagy by blocking autophagosome formation via the inhibition of PI3KC3. 3-MA was resuspended in water because organic solvents such as DMSO display cytotoxicity. Due to its instability in aqueous solutions, 50 mM stocks were prepared freshly; cells were treated with 5 mM 3-MA to inhibit autophagy. Same final water concentrations were used for negative controls. 200 U/ml IFN-γ (Sigma Aldrich) was also dissolved in water and used as an inducer of autophagy in mycobacterial infection experiments^65^. The inhibitor and inducer of autophagy or combinations thereof were added to the cells 24 h ahead of infection (as well as during infection) and samples for RT-qPCR or immunostaining were taken at indicated time points. All experiments were performed at least in triplicate. Expression level of Beclin1 mRNA was observed by means of RT-qPCR (5′-caggagagacccaggaggaa-3′ and 5′-gctgttggcactttctgtgg-3′).

Autophagy was followed in infected cells by means of immunostaining along with fluorescently labelled BCG. For this purpose, THP-1 derived macrophages were fixated with 4% Roti-Histofix (Carl Roth) and immunostaining was performed as described previously^66^ using a 1:100 dilution of the antibody LC3A/B (D3U4C) XP® Rabbit mAb (Cell Signaling Technologies) together with the secondary antibody Goat-anti-Rabbit, DyLight 594, #35560 (Thermo Scientific) using a 1:200 dilution. Caspase 3 activation was studied as described previously^66^. Microscopic photographs were taken with a fluorescence inverted microscope DMI6000 B (Leica). DAPI staining (Sigma-Aldrich, Phalloidin Atto 488) was used to stain nuclei. 3D Deconvolution blind method was used with total iterations of 10, refractive index 1.52 and remove background checked. Experiments were repeated three times.

### MEG3 knockdown and detection of autophagy

For knockdown of MEG3, siRNA were custom designed and synthesised by Sigma Aldrich (5′-gacuuaaaccaaugcccua-3′). 10^6^ THP-1 cells were transfected using the Nucleofector Technology (Lonza) together with 100 pmol siRNA and Kit V, respectively. Experiments were repeated at least three times. RNA samples were taken after 24 h of incubation. Along with MEG3 quantification, mRNA expression of LC3B (5′-ccgcaccttcgaacaaagag-3′ and 5′-ttgagctgtaagcgccttct-3′) was also measured in the samples.

For infection experiments, 24 h post nucleofection THP-1 cells were incubated for 1 h with BCG at MOI of 10. Afterwards, non-phagocytised BCG were washed and cells were further incubated for 24 h. THP-1 were lysed in 500 μl ultra pure water and 100 μl of 10^−2^ diluted lysates were plated in triplicate on middlebrook agar plate for bacterial counting as described earlier^67^.

For detection of autophagy after MEG3 knockdown, THP-1 cells were washed with PBS and lysed in RIPA buffer supplemented with protease inhibitor cocktail (Cell Signaling Technology #5871 S). If needed, 100 nM Bafilomycin A1 (TOCRIS bioscience) was added 24 h after transfection and samples were taken after 3 hours. Protein concentrations were measured using a protein assay kit (2D quant, GE Healthcare). Cell lysates (10–15 μg protein/lane) were separated by SDS-PAGE (13.5% gels) and transferred to 0.45 μm polyvinylidene difluoride membranes (PVDF) (GE Healthcare) as described previously^68^. The membrane was blocked by blocking buffer (5% skim milk powder in TBST) for 60 min at room temperature and then incubated with rabbit anti-LC3A/B (1:1000, Cell Signaling Technology, 12741 S) or rabbit anti-p62 (1:1000, Cell Signaling Technology, 5114 S) overnight. mTOR activation was studied using the antibodies pan-P70-S6K (1:1000, Cell Signaling Technology #49D7), pho-P70-S6K (1:1000, Ser371, Cell Signaling Technology #9208), pho-P70-S6K (1:1000, Thr389, CST #108D2). After proper washing, membranes were incubated with anti-rabbit horseradish peroxidase-conjugated secondary antibody (1:2000, Cell Signaling Technology), and chemiluminescence was detected using western blotting reagents (Cell Signaling Technology). After detection of LC3A/B or p62, membrane was stripped and again processed for GAPDH detection by using primary mouse mAb anti-GAPDH (1:10000, Abcam, ab8245) antibody and sheep anti-mouse secondary antibody (1:20000, GE Healthcare). Experiments were repeated at least three times.

For counting LC3 punctae, at least 3 randomly selected microscopic photographs of each biological replicate were analysed using ImageJ 1.48v^69^. Threshold value was adjusted carefully for strong autophagy signal and a particle size of 10–60 pixel^2^ for 1392 × 1040 resolution photograph was taken into consideration. Data is represented as average LC3 punctae per cell. An average of 12 cells per section were counted. For quantification of cellular bacterial loads after MEG3 knockdown, THP-1 cells were transfected as described above. After 24 h of incubation, they were infected with fluorescently labelled BCG at MOI of 10 as described above. After fixation, microphotographs were taken and the number of BCG per cell was counted manually. Triplicate experiments were performed. To prove the fluorescence based generation of bacterial counts, infected macrophages (three biological replicates) were lysed and CFU/ml were determined as described earlier^67^.

### Statistical and *in silico* analysis

Unpaired t tests were conducted applying GraphPad Prism version 6.00 for Windows, GraphPad Software, La Jolla California USA, www.graphpad.com. Asterisks in figures summarise P values (*P < 0.05; **P < 0.01; ***P < 0.001; ****P < 0.0001).

Heatmap and hierarchical clustering analysis were performed using the MultiExperiment Viewer (MeV) from the TM4 software package^70^. For this purpose, the fold differences of means (triplicate RT-qPCR measurements) were calculated and were subjected to MeV analysis. *In silico* analysis of lncRNA networks was performed by retrieving CLIP-Seq data based prediction of lncRNA-miRNA interactions using starBase v2.0^24^. Networks were extended by target analysis of miRNA lists using the CyTargetLinker^26^ together with Cytoscape^25^ and ClueGO analysis^28^ annotated functionally grouped gene ontologies and pathways. Following settings were considered for ClueGo analysis: significant KEGG pathways^29^ enrichment (P < 0.05), GO Term/Pathway Selection (3/4%), Kappa Score ≥ 0.3 and GO Term grouping.

## Additional Information

**How to cite this article**: Pawar, K. *et al.* Down regulated lncRNA MEG3 eliminates mycobacteria in macrophages via autophagy. *Sci. Rep.* **6**, 19416; doi: 10.1038/srep19416 (2016).

## Supplementary Material

Supplementary Dataset 1

## Figures and Tables

**Table 1:** Selected lncRNA, ncRNA and conventional reference genes for RT-qPCR arrays.

Targets (lncRNA & reference genes)	Accession No.	Forward primer (5′-3′)	Reverse primer (5′-3′)	Amplicon Size (bp)	Tm (°C)	Slope	Y-Intercept	R^2^	Efficiency	Dyn. range
ANRIL (CDKN2B-AS1)	NR_003529.3	acccaggctggagtgtattg	tagtcccagctgctcaggtt	81	60	−3.384	30.149	0.999	97.457	8
AS UCHL1 (UCHL1-AS1)	NR_102709.1	gtcgtctgcccaaaactagc	aaggtggacaccagctcatc	124	60	−3.131	29.645	0.994	108.630	9
EGO (EGOT)	NR_004428.1	taatcagctcagggggtcac	atacccaattccctgccttc	133	60	−3.496	31.101	0.999	93.233	8
GAS5	NR_002578.2	agaaatgcaggcagacctgt	gcactctagcttgggtgagg	108	60	−3.394	30.804	1.000	97.082	8
H19	NR_002196.1	ttcaaagcctccacgactct	ctgagactcaaggccgtctc	101	60	−3.347	29.198	0.999	98.956	8
HOTAIRM1	GQ479958.1	gaagagcaaaagctgcgttc	cagacctctcgccagttcat	140	60	−3.511	29.864	0.998	92.669	8
RPS15AP25 (LETHE LIKE)	NG_011025.2	caggaacatccacaatgctaaa	cagtgtagccatgcttcatca	107	60	−3.612	28.697	0.999	89.161	8
MEG3	NR_003530.2	cagccaagcttcttgaaagg	ttccacggagtagagcgagt	103	60	−3.400	27.506	0.999	96.838	8
NEAT1	NR_028272.1	gtggctgttggagtcggtat	attcactccccaccctctct	113	60	−3.427	29.274	0.998	95.806	9
NRON	NR_045006.1	ccccatttcacagaggaaga	ttgggccttctttacaccac	89	60	−3.448	30.652	0.999	95.010	8
NTT	U54776.1	ggtctccttaagggcaaagg	ccttgagaggccatttggta	86	60	−3.292	27.021	0.998	101.256	9
PRINS	NR_023388.1	ggaatgtggcctgtgttctt	agggacaaccacatcaaagc	116	60	−3.396	30.633	0.999	97.007	9
PTENP1	NR_023917.1	agttccctcagccgttacct	aggtttcctctggtcctggt	135	60	−3.334	29.109	0.999	99.503	8
RNCR3 (LINC00599)	NR_024281	agtcgttgggctatgtggac	ggatctctgcctgaaactgc	140	60	−3.490	26.643	0.999	93.430	8
TMEVPG1 (IFNG-AS1)	Ak124066	aaacgctggaggagaagtca	gttagcagttggtgggcttc	77	60	−3.311	27.708	0.998	100.466	8
TNCRNA (NEAT1)	AF080092	gacttggggatgatgcaaac	tcacaacagcatacccgaga	94	60	−3.397	28.417	1.000	96.944	8
WT1-AS	NR_023920	cctgagctaagcaccaggac	gtacaggagagcgcctatcg	85	60	−3.556	28.974	0.999	91.066	8
ZEB2NAT	HG494679	gaccgttattcctgcagagc	ccctccccacaaagataggt	73	60	−3.321	28.383	0.994	100.055	8
SNORD47	NR_002746.1	tgatgtaatgattctgccaaatg	acctcagaatcaaaatggaacg	77	60	−3.43	28.654	0.999	95.699	8
ACTB	NM_001101.3	ggacttcgagcaagagatgg	agcactgtgttggcgtacag	234	60	−3.295	31.373	1	101.1128	7
B2M	NM_004048.2	gtgctcgcgctactctctct	ggatggatgaaacccagaca	135	60	−3.577	32.358	1	90.369	7
GAPDH	NM_001256799.2	ccatcttccaggagcgagat	ctaagcagttggtggtgcag	249	60	−3.36	36.635	0.999	98.435	7
18 S rRNA	NR_003286.2	atggccgttcttagttggtg	cgctgagccagtcagtgtag	217	60	−3.34	32.878	1	99.264	7
